# Influence of treatment access on survival of metastatic renal cell carcinoma in brazilian cancer center

**DOI:** 10.1590/S1677-5538.IBJU.2020.0443

**Published:** 2020-12-20

**Authors:** Luciana de M. Leite, Paulo G. Bergerot, Aldo L. A. Dettino, José Augusto R., Stenio de C. Zequi, Maria Nirvana da C. Formiga

**Affiliations:** 1 AC Camargo Cancer Center Departamento de Oncologia Médica São PauloSP Brasil Departamento de Oncologia Médica, AC Camargo Cancer Center, São Paulo, SP, Brasil; 2 City of Hope Comprehensive Cancer Center Department of Medical Oncology and Experimental Therapeutics DuarteCA USA Department of Medical Oncology and Experimental Therapeutics, City of Hope Comprehensive Cancer Center, Duarte, CA, USA; 3 AC Camargo Cancer Center Departamento de Urologia Oncológica São PauloSP Brasil Departamento de Urologia Oncológica, AC Camargo Cancer Center, São Paulo, SP, Brasil; 4 Instituto Nacional de Ciência e Tecnologia em Oncogenômica e Inovação Terapêutica AC Camargo Cancer Center São PauloSP Brasil Instituto Nacional de Ciência e Tecnologia em Oncogenômica e Inovação Terapêutica, AC Camargo Cancer Center, São Paulo, SP, Brasil

**Keywords:** Carcinom, Renal Cell, Therapeutics, Kidney Neoplasms

## Abstract

**Background:**

Tyrosine kinase inhibitors (TKI) and immunotherapy improved survival in metastatic renal cell carcinoma (mRCC). Disparities in treatment access are present in healthcare systems globally. The aim of this study was to analyze survival outcomes of mRCC patients treated with first-line TKIs in the public (PHS) and private (PrS) health system in a Brazilian Cancer Center.

**Materials and Methods:**

Records from all mRCC patients treated with first-line TKIs from 2007-2018 were reviewed retrospectively. Categorial variables were compared by Fisher's exact test. Survival was estimated by Kaplan-Maier method and survival curves were compared using the log-rank test. Prognostic factors were adjusted by Cox regression model.

**Results:**

Of the 171 eligible patients, 37 (21.6%) were PHS patients and 134 (78.4%) were PrS patients. There were no difference in age, gender, or sites of metastasis. PHS patients had worse performance status (ECOG ≥2, 35.1% vs. 13.5%, p=0.007), poorer risk score (IMDC poor risk, 32.4% vs. 16.4%, p=0.09), and less nephrectomies (73% vs. 92.5%, p=0.003) than PrS patients. Median lines of therapy was one for PHS versus two for PrS patients (p=0.03). Median overall survival (OS) was 16.5 versus 26.5 months (p=0.002) and progression-free survival (PFS), 8.4 versus 11 months (p=0.01) for PHS and PrS patients, respectively. After adjusting for known prognostic factors on multivariate analysis, PHS patients still had a higher risk of death (HR: 1.61, 95% CI: 1.01-2.56, p=0.047).

**Conclusion:**

Patients with mRCC treated via the PHS had worse overall survival, possibly due to poorer prognosis at presentation and less drug access.

## INTRODUCTION

Kidney cancer is the third most common urologic malignancy, with 10.688 new cases estimated for 2018 in Brazil (1), with increasing incidence trends (2). Worldwide, up to 30% of patients have metastatic disease at the time of diagnosis, and another 20% will relapse at distant sites following nephrectomy (3).

The introduction of targeted therapies, such as tyrosine kinase inhibitors (TKI), and recently immunotherapy, has led to significant survival benefits for metastatic renal cell carcinoma (mRCC) patients treated in the first- (4–6) and second-line (7–9) settings, with extensive evidence from randomized clinical trials and meta-analyses (10). Beyond that, there is data to support that patients that are exposed to latter lines of therapy also have benefit in overall survival (11). Nevertheless, in Brazil, there are no approved second and further lines of treatment for mRCC in the Public Health System (PHS).

The Brazilian population has two main ways to access health services. One way is through a unified and universal PHS that is government funded and regulated. The other is through private insurance companies that are payed from out-of-pocket. In October 2018, private insurance coverage comprised of only 24.3% of Brazil's total inhabitants, with declining numbers over the last couple of years.

Hence, the objectives of this study were to evaluate differences in the clinical characteristics of mRCC patients treated under the PHS or private health systems (PrS) in a specialized Cancer Center in Brazil and to explore potential factors that influence survival outcomes in these two groups. The main hypothesis was that PHS patients would have worse overall survival than PrS patients.

## MATERIALS AND METHODS

### Study Design and Participants

This study was designed as retrospective and was carried out at A.C. Camargo Cancer Center (ACCCC), a single center specialized in cancer treatment in Brazil. At ACCCC, healthcare is provided to patients with public and private insurance, but because it is a private institution, most patients (∼80%) have private insurance. The ACCCC database was searched for all mRCC patients who were treated with first-line TKIs from January 2007-January 2018. Patients who had fewer than three physician visits, who had a follow-up time <6 months before death or progression occurred, or for whom there was insufficient data regarding the first-line of treatment were excluded.

A total of 273 mRCC patients, including 224 PrS patients and 49 PHS patients, were screened. Ninety of the 224 PrS patients and 12 of the 49 PHS patients were excluded. The main reasons for exclusion in the PrS group were loss to follow-up/missing data and not receiving TKIs as the first-line of treatment. In the PHS group, the main reasons for exclusion was not receiving TKIs at the first-line of treatment and being treated with best supportive care only. The study diagram is shown in Supplemental Material 1.

**Supplemental Material 1:**
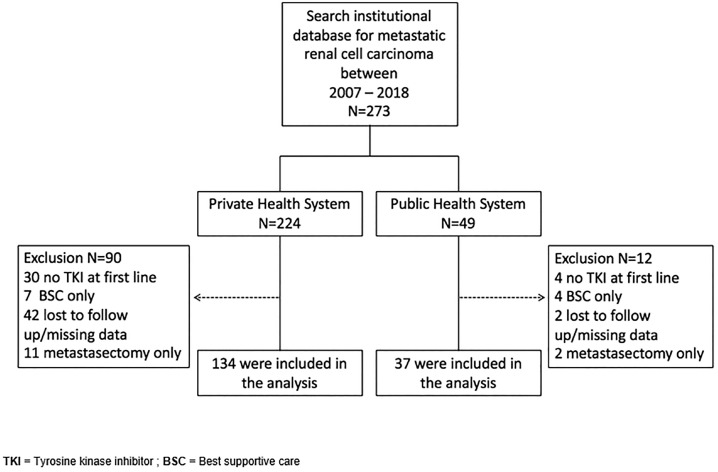
Study diagram.

The following baseline clinical and prognostic data were collected from medical records: age, gender, type of health coverage (PHS or PrS), number and sites of metastasis (12), performance status as defined by the Eastern Cooperative Oncology Group (ECOG) (13), and the International Metastatic Renal Cell Carcinoma Database Consortium (IMDC) risk model classification (14). Due to the small sample size, the ECOG variable was recorded as 0-1 versus ≥2.

Data were also collected on factors related to treatment, including the type and number of systemic agents received, time from the metastasis diagnosis and initiation of TKI, and surgical treatments received, including nephrectomy or metastasectomy (considered only if all metastatic lesions and the primary tumor have been surgically removed). This study was approved by the ACCCC Internal Ethics Review Board (number 2598/18).

### Treatment

The choice of TKI and the starting dose were determined by the attending physician. Sunitinib was prescribed orally at a dose of 50 milligrams (mg) per day on the standard 4 weeks on-2 weeks off schedule or on the 2 weeks on-1 week off schedule. Pazopanib was prescribed at 800mg per day, or in a reduced dose of 600mg daily. When sorafenib was prescribed, the dose was 800mg daily.

### Statistical Analysis

The clinical and demographic variables of the PHS and PrS patients were reported as relative and absolute frequencies. Fisher's Exact test and the Mann-Whitney test were used to identify significant differences in categorial and continuous variables, respectively.

The primary outcome of the study was overall survival (OS) according to patient health coverage (PHS versus PrS) and the secondary outcome was progression-free survival (PFS), also stratified by those two groups. OS was defined as the time from the start of first-line TKI to death (from any cause). PFS was defined as the time from the start of TKI treatment to death or disease progression (whichever occurred first). Disease progression was assessed based on the information in patient's charts as described by the attending physician. It is an institutional practice to assess disease progression by imaging every two or three months and to analyze the images according to RECIST 1.1 criteria (15). No central review of the imaging was done.

Survival curves were generated according to the Kaplan-Meier method and were compared using the log-rank test. Two-tailed P values <0.05 were considered statistically significant. Variables with Wald' P values <0.05 in the univariate analysis and that are known clinical prognostic factors for mRCC were selected for multivariate analysis. Statistical analysis was performed with the software SPSS 23.0 (SPSS, Chicago, IL).

## RESULTS

### Patients Characteristics

Between January 2007 and 2018, 171 eligible mRCC patients were treated at ACCCC with TKIs as the first-line treatment. The majority (134/171, 78.4%) of the patients had private insurance, and 37/171 (21.6%) received care via the PHS. Clinical characteristics according to type of health coverage and treatments received are described in Table-1.

**Table 1:** Baseline clinical characteristics and treatment by type of health coverage (private versus public funded).

Characteristics		Private n=134	Public n=37	p
Median age		61	61	0.78
(range)		(24-84)	(27-78)	
**Sex (%)**				
	Male	102 (76.1)	30 (81.1)	0.66
	Female	32 (23.9)	7 (18.9)	
**Histology (%)**				
	Clear cell	117 (87.3)	25 (67.6)	0.01
	Non-clear cell	17 (12.7)	12 (32.4)	
**ECOG (%)**				
	0-1	115 (86.5)	24 (64.9)	0.007
	≥2	18 (13.5)	13 (35.1)	
Missing		1		
**Metastasis (%)**				
	Synchronic	48 (35.8)	22 (59.5)	0.014
	Metachronic	86 (64.2)	15 (40.5)	
**IMDC risk (%)**				
	Favorable	34 (26.6)	6 (16.2)	0.09
	Intermediate	73 (57)	19 (51.4)	
	Poor	21 (16.4)	12 (32.4)	
	Missing	6		
Median No. metastatic sites (range)		2 (1-5)	2 (1-4)	0.99
**Metastasis sites (%)**				
	Lung	88 (65.7)	24 (64.9)	1.0
	Lymph nodes	66 (49.3)	20 (54.1)	0.71
	Bone	47 (35.1)	13 (35.1)	0.99
Median lines of therapy (range)		2 (1-5)	1 (1-4)	0.03
**Total lines of therapy received (%)**				
	1	52 (38.8)	22 (59.5)	0.096
	2	47 (35.1)	9 (24.3)	
	≥3	35 (26.1)	6 (16.2)	
Prior nephrectomy (%)		124 (92.5)	27 (73)	0.003
Metastasectomy (%)		30 (22.4)	6 (16.2)	0.5
**1**^st^ **line TKI (%)**				
	Sunitinib	90 (67.2)	34 (91.9)	0.008
	Pazopanib	40 (29.9)	3 (8.1)	
	Sorafenib	4 (3)	0	

**ECOG** = Eastern Cooperative Oncology; **IMDC** = International Metastatic Renal Cell Carcinoma Database Consortium risk model classification; **TKI** = Tyrosine kinase inhibitor

Between the PrS and PHS groups, there were no baseline differences in median age (p=0.78), gender (p=0.66), number of metastatic sites (p=0.99) and sites of metastasis (lung, p=1.0; lymph nodes, p=0.71; bone, p=0.99). However, PHS patients had a higher proportion ECOG performance ≥2 (p=0.007), poor IMDC risk (p=0.09), non-clear cell histology (p=0.01) and synchronic metastasis at initial diagnosis (p=0.014). There was also an imbalance in the proportion of nephrectomies (p=0.003) and metastasectomies (p=0.5) performed, with PHS patients having fewer of these procedures than PrS patients. No difference was seen in the median time from the diagnosis of metastatic disease to the beginning of the TKI treatment (2.29 versus 1.79 months, p=0.59).

### Treatment and efficacy outcomes

Sunitinib was the most common choice of first-line agent in both groups, being prescribed for 91.9% and 67.2% of the PHS and PrS patients, respectively. Pazopanib and sorafenib were also used as shown in Table-1. The median number of treatment lines employed was one for PHS patients versus two for PrS patients (p=0.03).

**Figure 1:**
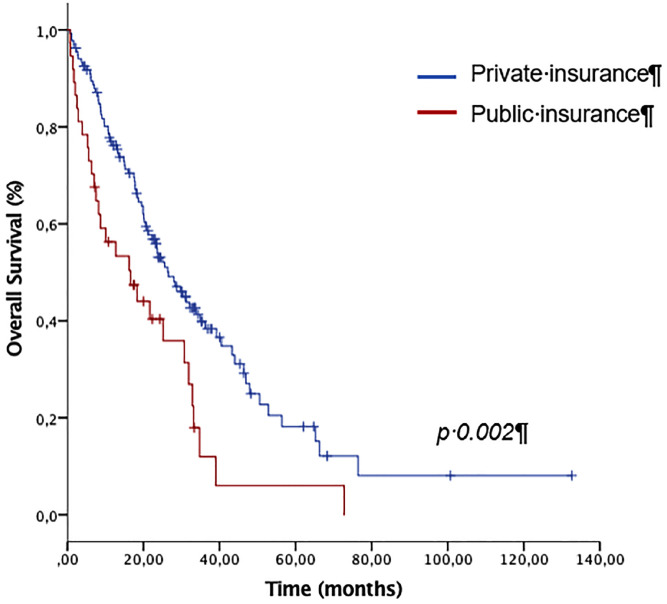
Overall survival by type of health coverage (private versus public).

With a median follow-up of 35.4 months (95% CI: 31.4-39.3 months), the overall survival was 16.5 months for PHS patients versus 26.5 months for the PrS patients (p=0.002; Figure-1). Progression-free survival was 8.4 months versus 11 months (p=0.01), for the PHS and PrS patients, respectively.

Multivariate analysis for overall survival was performed, with the objective to adjust the type of health coverage for other known clinical prognostic factors that were statistically significant on the univariate model and were imbalanced at baseline. After this analysis, PHS patients still had a higher mortality (HR: 1.61, 95% CI: 1.01-2.56, p=0.047). The results of the univariate and multivariate analyses are shown in Table-2. In addition to the type of health coverage, having poor prognostic risk by the IMDC score (p <0.001), an ECOG ≥2 (p=0.04), and two or more sites of metastasis (p <0.001) were also significant adverse factors for OS in the multivariate model.

**Table 2:** Univariate and Multivariate analysis for overall survival.

Variable	Univariate (95% CI)	P	Multivariate (95% CI)	P
Type health coverage (public)	1.92 (1.25-2.94)	0.003	1.61 (1.01-2.56)	0.047
No prior nephrectomy	2.47 (1.46-4.17)	0.001	1.21 (0.64-2.29)	0.56
IMDC poor risk	3.80 (2.37-6.06)	<0.001	3.15 (1.79-5.53)	<0.001
ECOG ≥2	3.43 (2.15-5.45)	<0.001	1.81 (1.02-3.22)	0.04
Synchronic metastasis	1.56 (1.07-2.26)	0.02	1.32 (0.86-2.03)	0.21
≥2 sites metastasis	2.02 (1.28-3.20)	0.0030	2.81 (1.67-4.71)	<0.001
Non-clear cell histology	1.51 (0.94-2.44)	0.09		

**ECOG** = Eastern Cooperative Oncology; **IMDC** = International Metastatic Renal Cell Carcinoma Database Consortium risk model classification; **95% CI** = 95% Confidence Interval

Regarding treatment-related effects on survival, for the whole cohort, receiving two or more lines of treatment was a significant prognostic factor, with a median OS of 31.2 months versus 12.7 months in favor of those patients who received multiple lines of treatment (HR: 0.51, 95% CI: 0.35-0.75, p=0.001). Furthermore, when this factor was inserted into the previous multivariate model in an exploratory analysis, the type of health coverage lost its statistical significance, leading to the hypothesis that this was one of the important drivers and confounders of the survival differences observed between the study groups (Supplemental Material 2).

**Supplemental Material 2:** Univariate and multivariate OS analysis after including the number of lines of treatment.

Variable	Univariate (95% CI)	p	Multivariate (95% CI)	p
Type health coverage (public)	1.92 (1.25-2.94)	0.003	1.59 (0.99-2.55)	0.057
No prior nephrectomy	2.47 (1.46-4.17)	0.001	1.02 (0.53-1.96)	0.96
IMDC poor risk	3.80 (2.37-6.06)	<0.001	2.74 (1.55-4.85)	0.001
ECOG ≥2	3.43 (2.15-5.45)	<0.001	1.97 (1.12-3.46)	0.02
Synchronic metastasis	1.56 (1.07-2.26)	0.02	1.31 (0.82-2.09)	0.26
≥2 sites of metastasis	2.02 (1.28-3.20)	0.003	2.80 (1.65-4.73)	<0.001
≥2 lines of treatment	0.51 (\0.35-0.75)	0.001	0.51 (0.34-0.77)	0.001

**ECOG** = Eastern Cooperative Oncology; **IMDC** = International Metastatic Renal Cell Carcinoma Database Consortium risk model classification; **95% CI** = 95% Confidence Interval

## DISCUSSION

To our knowledge this is the first dataset that directly compares survival outcomes of Brazilian mRCC patients treated via public or private health systems. A previous report of patients treated in a public cancer center in Brazil found that the median OS was 15.2 months for PHS patients treated with sunitinib as the first-line agent and 14.2 months for PHS patients treated with pazopanib as the first-line agent (16). Similarly, in this study, the median OS was 16.5 months for PHS patients.

Some known prognostic factors for mRCC differed between the two groups (17, 18). The inferior PFS observed in PHS patients could be due to a higher proportion of adverse baseline characteristics, such as poor IMDC risk, ECOG performance ≥2, synchronic metastasis, and non-clear cell histology (although non-clear cell histology was not statistically significant in this cohort).

The IMDC risk model has been extensively validated and is used to estimate survival with targeted therapies (19). One hypothesis for the higher incidence of poor IMDC risk in the PHS patients may be delays in diagnosis and reduced access to specialized cancer centers, resulting in more advanced disease stages at the beginning of treatment. Bergerot (20) also found the same disparities regarding distribution of poor IMDC risk in PHS patients upon evaluation of treatment patterns for Brazilian mRCC patients using data retrieved from a commercial database that contained information from public and private hospitals. Of 3.149 patients who received first-line therapy, only 641 (20%) were given a second line agent. Those differences were more profound when comparing patients treated in private or public hospitals, were 14% versus 7% received a second-line therapy (p=0.001).

In this study, having less access to further lines of treatment was linked to detrimental survival outcomes in the PHS population. Access to cancer drugs and the increasing expenses associated with new therapies are a challenge worldwide, but are especially difficult to overcome in lower and middle income countries, where issues like fragmentation of health systems, delays in approval by local regulatory agencies and underfunding add to the challenge (21).

The disparities observed are expected to be further deepened because major shifts in the first-line treatment for clear cell mRCC occurred after 2018. Currently, the combination of nivolumab and ipilimumab is the standard of care for intermediate and poor IMDC risk, and the TKI-immunotherapy combo of axitinib and pembrolizumab is also a recommended option in guidelines, for all IMDC risk groups (22). However, single agent TKIs may still play a role in favorable IMDC risk, with comparable results for sunitinib versus axitinib plus pembrolizumab in this subgroup (23).

The main limitations of this study are the retrospective nature and small sample size. However, its strengths come from the use of real life data to show how fragilities in primary care, involving cancer diagnosis and referencing, and access to life prolonging cancer drugs can affect OS of patients with mRCC, even in a specialized cancer center were access to surgery, radiotherapy, hospital infrastructure and medical expertise are the same regardless of type of insurance. Exposing the fragilities in the PHS is the first step to tackle the problem and develop strategies to improve the public guidelines for the treatment of mRCC.

## CONCLUSION

Patients with metastatic renal cell carcinoma treated in the public health system had significantly worse overall survival, possibly due to poorer prognosis at presentation and less drug access.
